# Inhibitory activities of propolis, nisin, melittin and essential oil compounds on *Paenibacillus alvei* and *Bacillus subtilis*

**DOI:** 10.1590/1678-9199-JVATITD-2022-0025

**Published:** 2022-09-12

**Authors:** Alessandra Aguirra Sani, Ana Flávia Marques Pereira, Alessandra Furlanetto, Débora Silva Marques de Sousa, Tatiane Baptista Zapata, Vera Lucia Mores Rall, Ary Fernandes

**Affiliations:** 1 Department of Chemical and Biological Sciences, Botucatu Biosciences Institute (IBB), São Paulo State University (UNESP), Botucatu, SP, Brazil.

**Keywords:** Sporicidal activity, Apis mellifera, Cinnamaldehyde, Eugenol, Melittin, Nisin, Synergism, Tetracycline

## Abstract

**Background:**

Natural products represent important sources of antimicrobial compounds. Propolis and compounds from essential oils comprise good examples of such substances because of their inhibitory effects on bacterial spores, including bee pathogens.

**Methods:**

Ethanol extracts of propolis (EEP) from *Apis mellifera* were prepared using different methods: double ultrasonication, double maceration and maceration associated with ultrasonication. Together with the antimicrobial peptides nisin and melittin, and compounds present in the essential oils of clove (*Syzygium aromaticum*) and cinnamon (*Cinnamomum zeylanicum*), assays were carried out on one *Bacillus subtilis* isolate and *Paenibacillus alvei* (ATCC 6344) against vegetative and sporulated forms, using the resazurin microtiter assay. Synergism with all the antimicrobials in association with tetracycline was verified by the time-kill curve method. Potassium and phosphate efflux, release of proteins and nucleic acids were investigated.

**Results:**

EEPs showed the same MIC, 156.25 µg/mL against *B. subtilis* and 78.12 µg/mL against *P. alvei*. The peptides showed better activities against *B. subtilis* (MIC of 12 µg/mL for melittin and 37.50 µg/mL for nisin). Antimicrobials showed similar inhibitory effects, but cinnamaldehyde (39.06 µg/mL) showed the best action against *P. alvei*. Melittin and nisin showed the greatest capacity to reduce spores, regarding *B. subtilis* there was a 100% reduction at 6.25 and 0.78 µg/mL, respectively. Concerning *P. alvei*, the reduction was 93 and 98% at concentrations of 80 µg/mL of melittin and 15 µg/mL of nisin. EEPs showed the highest effects on the protein release against *B. subtilis* and *P. alvei*. Nucleic acid release, phosphate and potassium efflux assays indicated bacterial cell membrane damage. Synergism between antimicrobials and tetracycline was demonstrated against both bacteria.

**Conclusion:**

All antimicrobials tested showed antibacterial activities against vegetative and sporulated forms of *P. alvei* and *B. subtilis*, especially nisin and melittin. Synergism with tetracycline and damage on bacterial cell membrane also occurred.

## Background

The *Paenibacillus* species was originally included in the genus *Bacillus* according to its morphological characteristics common to *Bacillus subtilis*, which was isolated in 1872. The classification was based on the rod-shaped form, being aerobic or facultative anaerobic and forming spores. However, these characteristics are no longer suitable for grouping species into a single genus [1]. 

Some Paenibacillaceae species are specialized insect pathogens, such as *Paenibacillus lentimorbus* and *Paenibacillus popilliae*, the causative agents of milky disease in scarab beetle grubs, and *Paenibacillus larvae*, the causative agent of American foulbrood (AFB) in honeybees (*Apis mellifera*) [1]. Some *Paenibacillus* species are also opportunistic infectants for humans as well as causing spoilage of pasteurized dairy products [2]. 

*Paenibacillus alvei (P. alvei)*, *Enterococcus faecalis (E. faecalis)*, *Enterococcus faecium (E. faecium)*, *Achromobacter eurydice (A. eurydice)* are also frequently isolated in honeybee larvae when hives are infected with *Melissococcus pluton*, considered the causative agent of European foulbrood (EFB). In Australia, *P. alvei* was the third most common isolate in hives of *A. mellifera*, whereas *E. faecalis*, *E. faecium*, and *A. eurydice* were rarely isolated in hives affected by EFB. In addition, *P. alvei* can also produce signals in *A. mellifera* larvae similar to those produced by *Paenibacillus larvae* in AFB disease [3]. 

In EFB, the brood dies between the 3^rd^ and 5^th^ day of the larval stage. These bacteria survive up to three years in the dry remains of dead larvae. In AFB, the death in the pre-pupa stage, or young pupa, results in yellowish to brown color of the body, with a viscous consistency and a strong smell [4]. 

*Bacillus subtilis* is an important spore-forming bacterium with potential use as control agent against plant pathogens as well as biosurfactant [5]. Much of the knowledge about sporulation in bacteria was acquired by studying *B. subtilis*, which is considered a model organism in this genus [6]. 

Although antibiotics have revolutionized the treatment of infectious disease, the rapid adaptation of bacterial species, together with the intensive use of antimicrobial drugs, allowed the emergence and spread of resistant strains, making it urgent to search for new medicines with different mechanisms of action. Thus, natural products such as antimicrobial peptides (AMPs), essential oils and propolis are promising alternatives [7-9]. 

Antimicrobial peptides (AMPs) are molecules of natural origin that present antimicrobial activity including against multidrug-resistant bacteria. They act on cell membranes, making the development of bacterial resistance less likely, in addition to possible additive or synergistic activity between AMPs and antimicrobial drugs [10]. 

Essential oils are mixtures of volatile compounds of plant origin with about 20-60 compounds, with two or three of these compounds present in relatively high concentrations (20-70%) and therefore called major compounds [11]. They have antimicrobial activity and can act in synergism with propolis [9, 12].

Honeybees are important to humans not only because of pollination, but also due to the products that they produce. *Apis mellifera* is an important source of compounds with a variety of biological properties, especially propolis and melittin. The last is the main compound present in apitoxin (about 50% of the dry weight) that has antibacterial activity [7, 13-15].

Propolis is composed of a resinous material collected from exudates and plant shoots that is mixed with wax and enzymes. Its color and composition vary according to its origin and can contain more than 300 chemical compounds. Polyphenols and terpenoids are considered the most active compounds, whereas the flavonoid group includes chrysin, pinocembrin, apigenin, galangin, kaempferol, quercetin, tectochrysin, pinostrobin, and others [16]. 

The word propolis (*pro* = in defense and *polis* = city) suggests its importance for bees as it is used to coat internal walls, protect the colony against pathogens and temperature drop, cover carcasses of invaders who die inside the hive (to prevent their decomposition) and exhibits antibacterial, antioxidant, anti-inflammatory activities and other biological properties [4, 7, 17].

Melittin is an amphipathic peptide that has cytolytic activity, with action on the cell membrane by interfering with lipid molecules and with the formation of pores and the consequent permeabilization and exit of ions, water, and metabolites, causing cell death [18-20]. 

 The action of melittin varies according to the composition and concentration of lipids in the membrane as well as the temperature and the concentration of the melittin present, making it impossible to conclude about the mechanism that causes membrane rupture and whose molecular processes are responsible for this rupture [21, 22]. 

Melittin stands out both for its antibacterial action, including biofilm formation and synergism with antibacterial drugs [23]. Pereira et al. [24] reported the synergistic effect of melittin with oxacillin on methicillin-resistant *Staphylococcus aureus* (MRSA), with a minimum inhibitory concentration (MIC) value of around 7 µg/mL.

Nisin is an antimicrobial peptide produced by *Lactococcus lactis* with an action mechanism of pore formation in the cytoplasmic membrane as well as action on bacterial spores [25, 26]. It has synergism with oxacillin, thus minimizing possible toxicity considering that it will be possible to reduce therapeutic doses for the use of antimicrobial drugs and the emergence of bacterial resistance [27]. 

Cinnamaldehyde is the major compound of cinnamon (*Cinnamomum zeylanicum*) essential oil. Its antimicrobial activity has been widely reported, as well as its anti-inflammatory, angiogenic, and healing action [28]. Among its mechanisms of action are changes in the cell membrane by altering the lipid profile, inhibition of ATPase, inhibition of cell division, inhibition of gram-negative wall porins, inhibition of motility, inhibition of quorum sensing and biofilm formation, as well as synergistic action with antibiotics [29]. 

Eugenol is the major compound of clove essential oil (*Syzygium aromaticum*) and has antimicrobial, antioxidant, anti-inflammatory, anesthetic, anticancer, antiulcerogenic, anthelmintic, antipyretic, antidepressant, antistress, antispasmodic and relaxing activities, is also helps bone preservation, enhance skin permeation, and presents insecticidal and pest control actions [30, 31], which makes it of great interest to the cosmetic and pharmaceutical industry. The mechanisms of action for its antibacterial activity are cell membrane disruption as well as activity against some bacterial enzymes such as protease, histidine carboxylase, amylase, and ATPase [32]. 

The objective of the present study was to verify the antibacterial and sporicidal activity of ethanolic extracts of propolis (EEP), antimicrobial peptides (melittin and nisin), cinnamaldehyde and eugenol against *Paenibacillus alvei* and *Bacillus subtilis*, to demonstrate synergism of all the antimicrobials with tetracycline and verify their possible action on the bacterial cell membrane.

## Methods

### Propolis sample and ethanolic extract of propolis (EEP)

One sample of propolis was obtained from an apiary in the Botucatu city - São Paulo state, Brazil (22^o^55”10’ S e 48^o^29”35’ O) - containing swarms of *Apis mellifera* bees, kept in standard Langstroth hives, with wild vegetal sources. Propolis was collected in November 2018 by scraping it from the internal parts of the hives [33]. 

Ethanol extracts of propolis (EEP) were obtained using three different methods of alcoholic extraction: ultrasonication-ultrasonication (U+U), maceration-maceration (M+M), and maceration-ultrasonication (M+U), a methodology modified from Escriche and Juan-Borrás [34]. Each extraction method was carried out in duplicate and followed by the mixture of both extracts, which were used in the microbiological tests as well as the respective chemical characterizations.

EEP U+U: 7.5 grams of crude propolis was dissolved in 25 mL of 70% ethanol, followed by the first ultrasonication in an ultrasonic bath at 25 ^o^C for 30 minutes. The extract was then filtered and the residue of this filtration was added to another 25 mL of 70% ethanol. The extraction process was repeated (second ultrasonication) under the same conditions. Both filtrates (1^st^ and 2^nd^ ultrasonication) were mixed and made up to 50 mL with 70% ethanol.

EEP M+M: 7.5 grams of crude propolis was dissolved in 25 mL of 70% ethanol, and this mixture was kept under constant stirring for 24 hours at room temperature and protected from light. The extract was filtered, and the residue from the first maceration received again 25 mL of 70% ethanol. The procedure of the first maceration was repeated, obtaining a second filtrate after 24 hours. Both filtrates (1^st^ and 2^nd^ maceration) were mixed and made up to 50 mL with 70% ethanol. 

EEP M+U: The first extraction (7.5 grams in 25 mL of 70% ethanol) was performed as described for the maceration method. The extract was subsequently subjected to a second extraction as described for the ultrasonication method. Again, both filtrates (1^st^ and 2^nd^ extraction) obtained in the two ways described were mixed and made up to 50 mL with 70% ethanol.

We emphasize that the three forms of preparation allowed us to calculate that at the end of the preparation, the EEPs were considered 15% (15 grams/100 mL of 70% ethanol). The dry weight was determined after complete evaporation of propolis solvent in an aliquot (1 mL), obtaining 82.0, 82.1, and 115.7 mg/mL, respectively, for U+U, M+M, and M+U. The EEPs were preserved at 4 ^o^C until determination of total phenolic, flavonoid, and antioxidant activities, as well as microbiological assays.

### Nisin, melittin and compounds from essential oils

Melittin (approximately 65% purity), from *Apis mellifera* apitoxin, and nisin (approximately 2.5% purity) from *Lactococcus lactis* were purchased from Sigma-Aldrich^®^ (Merck^™^) and the compounds from essential oils were cinnamaldehyde (density 1.05 g/mL) and eugenol (1.06 g/mL), also from Sigma-Aldrich^®^ (Merck^™^).

### EEP chemical analysis and antioxidant action

The total phenolic compound analysis of the EEPs was performed by the Folin-Ciocalteu spectrophotometric method as described by Woisky and Salatino [35], using gallic acid as standard. Absorbance was measured in a spectrophotometer (UV Mini-1240) at 740 nm, and the results are expressed in gallic acid equivalents (mg/g). Total flavonoids were quantified by colorimetric reaction, using a mixture of 0.5 mL of ethanolic propolis extract, 4.3 mL of 80% ethanol, 0.1 mL of 10% aluminum nitrate, and 0.1 mL of 1 mol/L potassium acetate. After 40 minutes, absorbance was measured in a spectrophotometer at 425 nm, and the flavonoid content was expressed in quercetin equivalents (mg/g) as described in Park et al. [36]. 

The antioxidant activities of EEPs were determined by the evaluation of the DPPH radical scavenging activity (2,2-diphenyl-1-picryl-hydrazyl). The reaction mixture consisted of diluting 0.1 mL of each ethanolic extract of propolis and 0.4 mL of the extractor, 3 mL of ethanol, and 0.3 mL of the 0.5 mM solution of the DPPH radical, and after 45 minutes, an absorbance reading was taken at 517 nm.

### Bacterial strains

Microbiological assays were performed with *Bacillus subtilis* (soil isolate) and American Type Culture Collection (ATCC) standard *Paenibacillus alvei* number 6344.

Minimum inhibitory concentration (MIC) and minimum bactericidal concentration (MBC) by microdilution

The MIC values were determined using the microdilution methodology in 96-well microplates (Resazurin Microtiter Assay Plate - REMA) [37], testing product concentrations in Mueller Hinton broth. However, the growth of *P. alvei* was not adequate (incipient growth) in this medium. Then, brain heart infusion broth (BHI) was used. Suspensions (0.5 McFarland scale) of *P. alvei* and *B. subtilis* were prepared, followed by inoculation of the bacteria in the respective wells of the microplates at a concentration of around 10^5^ CFU/mL. The EEPs, compounds, and peptides were incubated with the bacteria; controls for 70% ethanol, dimethyl sulfoxide, and tetracycline [38, 39] were performed. The plates were incubated at 37 ºC for 24 hours in triplicate. After incubation, the MIC values were determined as the lowest concentrations of compounds capable of inhibiting bacterial growth. Turbidity was observed as soon after the addition of the resazurin redox revealing compound (0.05%), and growth was verified when the coloration changed from violet to pink.

Minimum bactericidal concentration (MBC) values were obtained in subcultures from the concentrations tested in microdilution assays, using BHI agar plates incubated at 37 ºC for 24 hours, with MBC being the lowest concentration in which there was no formation of colonies [40]. 

Spore suspensions and spore inhibitory action tests

Spore suspensions were prepared according to the method proposed by Fan et al. [41], with modifications. *Bacillus subtilis* and *P. alvei* were seeded on plates containing sporulation medium [10 g of peptone, 3 g of meat extract powder, 5 g of potassium chloride (KCl), 15 g of agar, and 0.2 g of manganese chloride (MnCl^2^) per liter and maintained at 37 °C/10 days]. Subsequently, the colonies were scraped off the surface of the medium using a sterile glass rod and suspended in sterile distilled water. The purification of the spores for elimination of the remaining vegetative cells was obtained by heating (80 °C for 20 minutes) the spore suspension, centrifuging (3300 g and 4 °C for 15 minutes) and washing it six times with sterile distilled water. The obtained spore pellets were resuspended in sterile distilled water and sonicated for 1 minute in an ultrasonic bath, followed by plating and counting. The purified suspension was then stored in an amber bottle at 4 °C until use in the assays.

Assays on spore action were performed by adding EEPs, compounds, peptides, and tetracycline in contact with spore suspensions, using an adaptation of the microdilution assay in 96-well microplates, with product concentrations obtained directly in sterile distilled water. The spore suspensions were standardized (around 10^4^ spores per mL), followed by the addition of spores at their respective concentrations, and kept under stirring at room temperature for 24 hours. The same controls used in the REMA assays were used, and all tests were performed in triplicate. 

To verify the action on spore counts, subcultures of the concentrations tested were prepared on BHI agar plates inoculated with 10 μL of each treatment, followed by incubation at 37 ºC for 24 hours. The colonies were then counted, and the respective Log of CFU/mL values obtained. An initial count was performed at the time of the addition of spores in the concentrations with the antibacterial products to compare spore counts before and after the experiment.

Synergism with tetracycline by the time-kill curve

The assays were carried out to obtain the survival curves (time-kill curve), with the aim to observe synergistic interactions between tetracycline with the different EEPs, compounds, and peptides in the respective MIC values and in combinations at 25% of the MIC of treatment + 25% MIC of tetracycline. The same controls of the other experiments were added. 

Synergism was evaluated on the bacteria standardized on the McFarland 0.5 scale and inoculated with 10^6^ CFU/mL. The culture medium used was BHI, and the microplates were incubated at 37 ^o^C for 24 hours in a microplate reader (Epoch 2, BioTek - 600 nm). These experiments were performed in triplicate and the results used to obtain the respective time-kill curve and synergism analysis as well as to determine whether the synergism was bactericidal or bacteriostatic.

In parallel with this procedure by the OD600, the same treatments and controls were prepared, and at 0, 2, 4, 8, and 24 hours, aliquots of the cultures were taken and inoculated into BHI agar plates (subcultures for plate counting). After incubation at 37 ºC for 24 hours, colony-forming units (CFU/mL) were counted to obtain the time-kill curve using this methodology. The CFU/mL values were calculated and transformed into Log CFU/mL. The synergistic or antagonist effects were established according to the reduction or increase of 2 log CFU/mL, respectively, in bacterial counts over a 24-hour period; it was also considered bactericidal when a reduction of ≥ 3 log CFU/mL from the initial inoculum occurred [42]. 

Efflux of potassium and phosphate ions

The assays were carried out using a modified methodology proposed by Lee, Kim, and Shin [43], aiming to verify the possible interference of the treatments on the permeability and disruption of the bacterial plasma membrane. Quantification of the concentrations of phosphate and potassium ions for both bacteria was performed with the Potassium test kit MQuant^®^ and the Phosphate test kit MQuant^®^ according to the manufacturer's protocol, using the time intervals of zero (T0) and after 4 hours (T4), in triplicate. The bacteria were standardized and diluted in peptone water (8.5 g of sodium chloride, 1 g of peptone in 1 L of sterile distilled water) and subsequently subjected to 1 and 2 MIC concentrations as determined in the microdilution assays.

Proteins release from cytoplasm

Protein release tests were performed to observe damage to the bacterial membrane because of the treatments carried out, and this release was quantified according to the concentration of proteins in the supernatant using the Bradford method [44]. 

The bacteria were inoculated in BHI medium and incubated at 37 ºC for 24 hours. Subsequently, the medium containing bacterial growth was separated into falcon tubes with treatments at 1 and 2 MIC from the microdilution assays, and analyses were carried out at T0 and T4. The samples were transferred to tubes and centrifuged at 10000 g, 4 ºC for 7 minutes, the supernatants were transferred with the reagent to a 48-well ELISA microplate and the OD was read at 595 nm. The experiments were performed in triplicate.

Cell membrane integrity

Cell membrane integrity was tested by the release of intracellular constituents [45]. Cells from 50 mL of *B. subtilis* and *P. alvei* cultures were collected after centrifugation (5000 g/15 min), washed three times, and resuspended in PBS. Subsequently, 40 mL of cell suspension was incubated at 37 ºC for 4 hours with the EEPs and compounds, including tetracycline, at concentrations of 1 and 2 MIC. After that, the suspension was centrifuged at 8000 g for 5 minutes, and the concentration of nucleic acids released in the supernatant was measured by UV absorption at 260 nm in a spectrophotometer at T0, T2, and T4. The experiments were performed in triplicate.

Statistical analysis

Data were submitted to one-way ANOVA, followed by Dunn's multiple comparison test. A P value ≤ 0.05 was considered a statistically significant difference. Analyzes were performed in GraphPad Prism 9.2^®^.

## Results

### Total phenolic, flavonoid, and antioxidant action

The total phenolic, flavonoid and antioxidant action (DPPH) are presented in Table 1. The EEP U+U showed the highest content for phenolic compounds (510.066 mg/100 mL) and an intermediate concentration in the flavonoid content (344.705 mg/100 mL). On the other hand, although the EEP M+U had a lower content of phenolic compounds (433.024 mg/100 mL), it had a higher content of flavonoids (391.764 mg/100 mL).

Both EEP M+M and EEP U+U were similar in terms of antioxidant activity and total phenolics. The EEP M+U extract showed a higher amount of flavonoid but lower values for total phenolic and antioxidant action, differing from the other EEPs.

**Table 1.** Chemical characterization of ethanolic extracts of propolis (total phenolic, flavonoid and antioxidant activity by DPPH).

	Total phenolic (mg/100 mL)	Flavonoid (mg/100 mL)	DPPH (mg/100 mL)
EEP U+U	510.066 ± 7.226^a^	344.705 ± 7.843^ab^	54.669 ± 0.697^ab^
EEP M+M	500.387 ± 14.195^ab^	302.875 ± 0.0^a^	55.061 ± 1.220^a^
EEP M+U	433.024 ± 2.581^b^	391.764 ± 2.614^b^	40.025 ± 0.348^b^

Different letters in the columns represent significant differences among distinct extraction methods when p ≤ 0.05.

### Minimum inhibitory concentration (MIC) and minimum bactericidal concentration (MBC)

The MIC and MBC values were identical for the three EEPs against *P. alvei* (78.12 µg/mL) and *B. subtilis* (156.25 µg/mL), with the exception of the EEP M+U with the highest MBC value (156.25 µg/mL) for *P. alvei* (Table 2). We also verified the lower sensitivity of *B. subtilis*. 

Melittin and nisin were the compounds with the highest effects on the growth of *B. subtilis*. For melittin, the MIC and MBC values were, respectively, 12.0 and 28.0 µg/mL, and for nisin, 37.50 µg/mL for both parameters. Cinnamaldehyde had the lowest MIC value (39.06 µg/mL), followed by melittin and nisin (50 µg/mL), against *P. alvei*. Eugenol showed inferior results against both bacteria (312.50 and 1250.0 µg/mL), obviously disregarding the values from ethanol assays. The tetracycline values shown below are lower than or equal to 4.68 µg/mL for bacteria in their vegetative state.

**Table 2.** Minimum inhibitory concentration (MIC) and minimum bactericidal concentration (MBC) (µg/mL) using the microdilution methodology (REMA) against *P. alvei* and *B. subtilis*.

	MIC (µg/mL)		MBC (µg/mL)	
	*B. subtilis*	*P. alvei*	*B. subtilis*	*P. alvei*
Tetracycline	0.58^e#^	2.34^d#^	0.58^e#^	4.68^d#^
Ethanol 70%	> 3950.00^b#^	> 3950.00^b#^	> 3950.00^b#^	> 3950.00^b#^
EEP U+U	156.25^a#^	78.12^a#^	156.25^a#^	78.12^a#^
EEP M+M	156.25^a#^	78.12^a#^	156.25^a#^	78.12^a#^
EEP M+U	156.25^a#^	78.12^a#^	156.25^a#^	156.25^ac#^
Nisin	37.50^c#^	50.00^a#^	37.50^d#^	50.00^a#^
Melittin	12.00^c#^	50.00^a#^	28.00^d#^	50.00^a#^
Eugenol	1250.00^d#^	312.50^a#^	2500.00^c#^	625.00^c#^
Cinnamaldehyde	312.50^ad#^	39.06^a#^	312.50^a#^	78.12^a#^

Different letters in the columns represent significant differences in antimicrobial activity between products for the same microorganism when p ≤ 0.05. Different symbols in the lines represent significant differences in the antimicrobial activity of the same product against different microorganisms when p ≤ 0.05.

### Sporicidal activities

The sporicidal activities (Table 3) are demonstrated by the highest percentage of spore reduction related to the lowest concentrations that promoted these reductions. Spores of *P. alvei* showed the greatest resistance to antimicrobials when compared to *B. subtilis*, especially when comparing the reductions for melittin and nisin, which were the most effective sporicidal agents against both bacteria. Tetracycline was less effective for the sporulated form, reducing 94% and 99% of *B. subtilis* and *P. alvei* spores, respectively, at a high concentration (300 µg/mL) when comparing the results against those found for vegetative bacteria. The sporicidal effects of cinnamaldehyde and eugenol were observed at higher concentrations (8000 and 6000 µg/mL, respectively).

**Table 3.** Reduction percentage in spore counting of *B. subtilis* and *P. alvei* that germinated 24 hours after contact with the compounds and their respective concentrations (µg/mL).

Treatments	*B. subtilis*		*P. alvei*	
	%	µg/mL	%	µg/mL
Tetracycline	94	300	99	300
Ethanol 70%	82	20000	33	20000
EEP U+U	83	6000	95	16000
EEP M+M	92	2000	79	12000
EEP M+U	86	8000	81	14000
Nisin	100	0.78	98	15
Melittin	100	6.25	93	80
Eugenol	87	6000	89	8000
Cinnamaldehyde	95	8000	87	8000

### Time-kill curve

**Figure 1.**
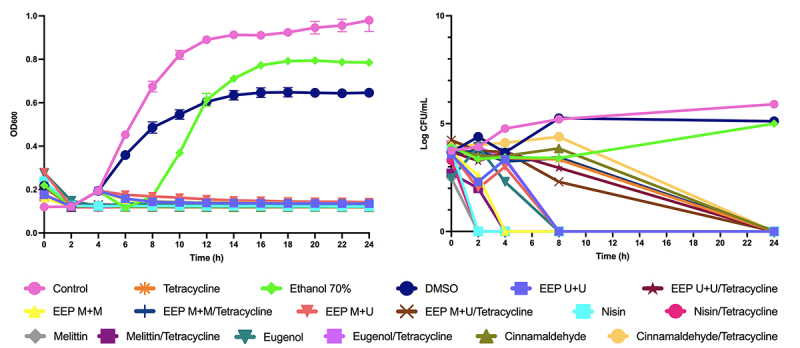
Time-kill curves from OD600 and Log of CFU/mL assays for EEPs, antimicrobial peptides, and major compounds and tetracycline tested alone and in combination with tetracycline on *B. subtilis*.

**Figure 2.**
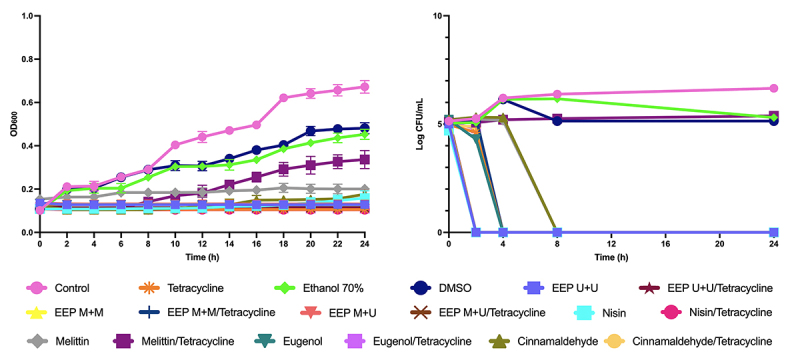
Time-kill curves from OD600 and Log of CFU/mL assays for EEPs, antimicrobial peptides, and major compounds and tetracycline tested alone and in combination with tetracycline on *P. alvei*.

The time-kill curves (OD and Log of CFU/mL) (Figures 1 and 2) showed that the EEPs and compounds associated with tetracycline had a synergistic effect with bactericidal or bacteriostatic action.

As seen in Figure 1, the combinations of nisin, melittin, and eugenol with tetracycline had synergism with the bactericidal effect for *B. subtilis* after 2, 4, and 8 hours, respectively, and the combinations of cinnamaldehyde, in addition to all EEPs, with tetracycline showed bactericidal effect after 24 hours.

For *P. alvei* (Figure 2), the combinations of EEP U+U, EEP M+U, and nisin with tetracycline also showed a bactericidal effect after 2 hours, and the combinations of EEP M+M, cinnamaldehyde, and eugenol with tetracycline were bactericidal after 4 hours. Melittin had no synergism with tetracycline by the OD600 methodology but showed a bacteriostatic effect by the Log CFU/mL methodology.

### Potassium and phosphate ions efflux

**Figure 3.**
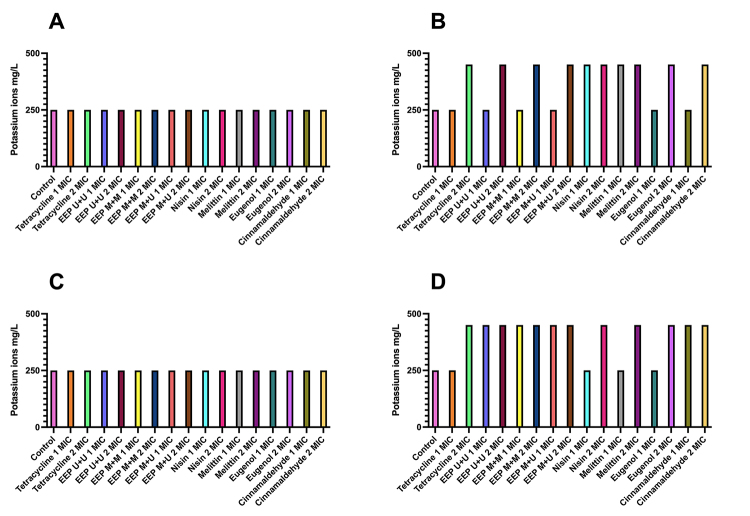
Release of potassium ions from *B. subtilis* at times **(A)** T0 and **(B)** T4 and *P. alvei* at times **(C)** T0 and **(D)** T4 after treatment with tested products at concentrations of 1 and 2 MIC.

The release of potassium ions (Figure 3) by *B. subtilis* was similar for the three EEPs, with release after 4 hours, always at a concentration of twice the MIC value, ranging from 250 to 450 mg/L. Melittin and nisin showed a release of potassium ranging from 250 mg/L at T0 to 450 mg/L at T4 considering both concentrations tested (1 MIC and 2 MIC).

For *P. alvei*, the EEPs showed similar results, ranging from 250 mg/L at T0 to 450 mg/L at T4, also at both concentrations. As for melittin, nisin, cinnamaldehyde, eugenol, and tetracycline, the release of potassium ions occurred after 4 hours only at a concentration of 2 MIC. 

**Figure 4.**
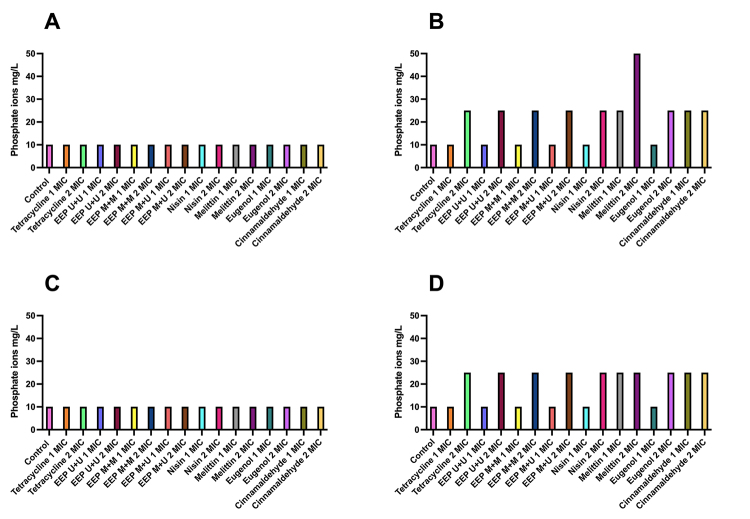
Release of phosphate ions from *B. subtilis* at times **(A)** T0 and **(B)** T4 and *P. alvei* at times **(C)** T0 and **(D)** T4 after treatment with tested products at concentrations of 1 and 2 MIC.

There were variations in the release and concentrations of phosphate ions against *B. subtilis* (Figure 4) for EEPs, nisin, and eugenol, from 10 mg/L in T0 to 25 mg/L in T4 at a concentration of 2 MIC. Melittin and cinnamaldehyde caused a release in T4 for concentrations 1 MIC and 2 MIC, with emphasis on melittin, which went from 10 mg/L in T0 to 50 mg/L in T4 when tested at a concentration of 2 MIC.

The EEPs, eugenol, nisin, and tetracycline showed a release of phosphate ions after 4 hours at 2 MIC, ranging from 10 mg/L at T0 to 25 mg/L at T4 against *P. alvei*. Melittin and cinnamaldehyde showed the best results, releasing 25 mg/L after 4 hours for both MIC concentrations. 

After 4 hours of exposure to the treatments, all products, even at different concentrations, released potassium and phosphate ions into the extracellular medium, demonstrating that the antimicrobials tested acted on the cell membrane.

### Protein release

**Figure 5.**
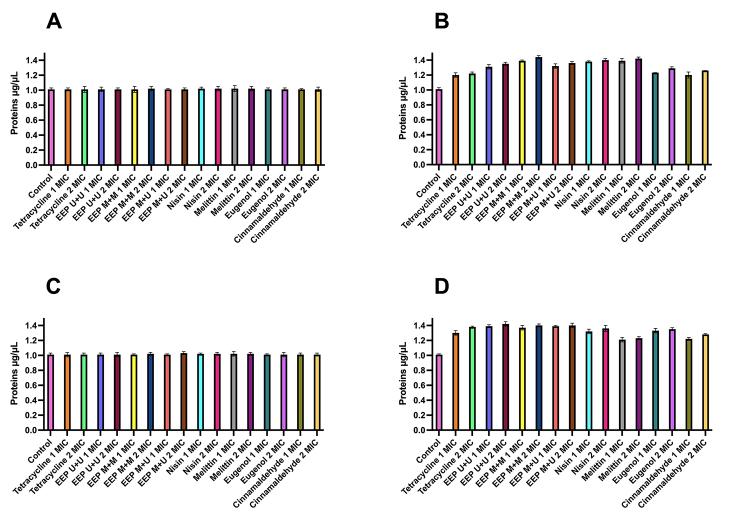
Proteins released in the supernatant (μg/μL) when exposed to concentrations equivalent to 1 and 2 MIC of the different treatments against *B. subtilis* at times **(A)** T0 and **(B)** T4 and against *P. alvei* at times **(C)** T0 and **(D)** T4.

The concentrations (μg/μL) of released proteins (Figure 5) were also tested at concentrations corresponding to 1 MIC and 2 MIC and at T0 and T4. The protein release gradually increased in all treatments.

The values of protein release on *B. subtilis* ranged from 1.20 to 1.44 μg/μL at T4, with the EEP M+M going from 1.02 μg/μL at T0 to 1.44 μg/μL in T4 at a concentration of 2 MIC, whereas for nisin and melittin, the concentrations ranged from 1.02 μg/μL (T0) to 1.40 μg/μL (T4) and 1.02 μg/μL (T0) to 1.42 μg/μL (T4), respectively, at 2 MIC.

For *P. alvei*, the variations ranged from 1.01 μg/μL in T0 to 1.42 μg/μL in T4, highlighting the EEPs, which varied from 1.01 μg/μL (T0) to 1.42 μg/μL (T4) for EEP U+U, 1.02 μg/μL (T0) for 1.40 μg/μL (T4) for EEP M+M, and 1.03 μg/μL (T0) for 1.40 μg/μL (T4) for EEP M+U, at a concentration of 2 MIC.

### Cell membrane integrity

**Figure 6.**
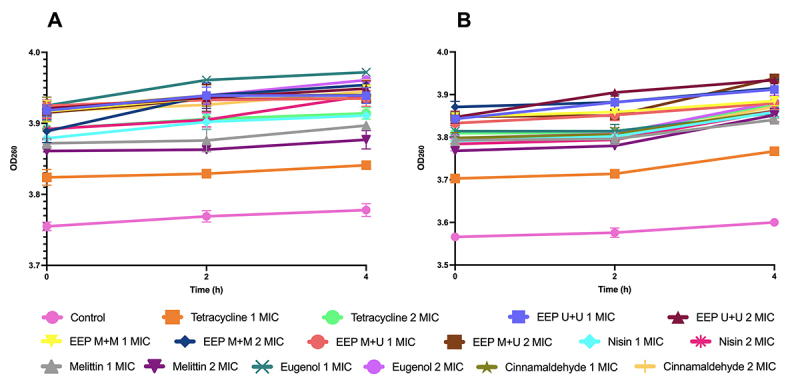
Absorbable cellular constituents at 260 nm released from **(A)** *B. subtilis* and **(B)** *P. alvei* after treatment with tested products at concentrations of 1 and 2 MIC.

The absorbable cellular constituents release (nucleic acid) at 260 nm (Figure 6), at T4 indicated damage to the bacterial cell membrane when exposed to the treatments, with emphasis on the action of the three EEPs against both bacteria, at a concentration of 2 MIC, with release values for EEP M+M of 3.88 (T0) to 3.95 (T4) for *B. subtilis* and EEP U+U and EEP M+U of 3.84 (T0) to 3.93 (T4) for *P. alvei*.

## Discussion

Due to the increased incidence of resistance by bacteria to conventional antimicrobial drugs [46], natural products such as propolis, antimicrobial peptides and essential oils are alternatives that aim to control bacterial growth, especially for *P. alvei* and others of same genus [47-50], and *B. subtilis* [51-54].

The EEPs showed similar inhibition effects for *B. subtilis* and *P. alvei*, without significant differences for the MIC obtained according to the preparation of the EEPs. In addition, there was no direct relation between the contents of total phenolic compounds and flavonoids and the MIC obtained. It was expected that a greater amount of these compounds would be directly related to antibacterial effects; however, the amount of these compounds is not always related to greater antibacterial activity [16]. In a previous study, Bridi et al. [55] suggested that other tests, such as the oxygen radical absorbing capacity (ORAC), in addition to the antimicrobial action tests themselves, should be considered for the establishment of international standards of propolis quality. Ghasemi et al. [56] also reported that the flavonoid content is not always related to antioxidant activity and that, depending on their structural conformation, some flavonoids may or may not have the ability to scavenge radicals.

The EEP U+U extraction method proved to be advantageous for being faster compared to other methods, without negatively influencing the respective antimicrobial activity. Escriche and Juan-Borrás [34] highlighted the speed of this EEP preparation as it requires around 1 hour for the complete process. Thus, extraction using an ultrasonic bath seems to be ideal when considering the time and yield of extraction, as well as the cost-effectiveness [57]. 

Melittin and nisin were the products with the higher inhibitory and bactericidal activities against *B. subtilis*, according to the low values of MIC and MBC obtained for this bacterium and above only those obtained with tetracycline. In addition, the results with nisin were similar to those with bee venom on *B. subtilis* [51, 58]. 

The antimicrobial activity of essential oils against *Paenibacillus larvae*, therefore the same genus as *P. alvei*, has been reported in the literature [48, 49], with similar results to those found in the present study. Cinnamaldehyde was the compound with the lowest MIC value against *P. alvei*.

When the two bacteria were compared, the same antimicrobial effects of each product were found, which is in agreement with the fact that the propolis, melittin, nisin, eugenol and cinnamaldehyde present action on bacterium cell membranes, including *B. subtilis* and *P. alvei* [19, 29, 32, 59, 60]. 

Spores of *P. alvei* showed greater resistance to the tested products when compared with *B. subtilis.* Most likely, exospore in *P. alvei* would be a factor for this tolerance to treatments, as this structure supposedly confers greater resistance to bacterial spores [61]. 

Sporulated bacteria play an important role in the evolution and dissemination of antibacterial drug resistance as they have a high capacity to resist treatments similar to those performed in this work. The ability of spores to remain at rest for a longer time in the environment allows these organisms to have a greater chance of undergoing genetic mutations and for the emergence of resistance to antimicrobial drugs, thus justifying the need for the high tetracycline concentration to reduce spore count [62]. Cinnamaldehyde and eugenol, also needed higher concentrations to present spore-reducing activity similar to that found by Alanazi et al. [54]. 

Melittin and nisin presented the major spore reductions on both bacteria. The ability of these peptides to form pores in membranes is known [59, 63]. Therefore, Gut et al. [64] suggest that the formation of pores in the membrane may also be fundamental for the inhibition of bacterial spores. Similarly, the interaction of nisin with lipid II, important in the formation of peptidoglycan in the bacterial wall, also exerts an effect on membrane integrity and inhibits cell differentiation from sporulated to vegetative form. Moreover, the activity of nisin against newly germinated spores is highlighted.

The spores evaluated were kept in deionized water until the tests were carried out, which may have induced pre-germination. This induction of germination may have made the spores more susceptible to treatment [65], thus justifying the lower concentrations of antimicrobial peptides needed to obtain spore inhibitory activity, highlighting the extremely low values of nisin against *B. subtilis* (0.78 µg/mL) and *P. alvei* (15 µg/mL).

The antibacterials tested demonstrated synergism with tetracycline, with bactericidal or bacteriostatic effects. Studies have demonstrated the synergistic activity of propolis, major compounds, and antimicrobial peptides with conventional antibiotics [10, 66, 67]. The synergistic potential among natural products with antibiotics may bring some benefits, such as the ability to potentiate the pharmacological effects and to prevent the selection of bacterial strains resistant to these same antibacterial drugs [68]. Thus, possibility of using tetracycline in association with the antibacterial products tested may be an advance in the knowledge and use of effective alternatives for the control of sporulated bacteria pathogenic to bees, mitigating the damage caused by *P. alvei*.

The treatments showed effects on the cell membrane of both bacteria. Bajpai et al. [69] described the relationship between the release of essential ions and absorbable cell constituents at 260 nm as a strong indication of a mechanism of antimicrobial action on the bacterial cell membrane and with irreversible damage. Essential oils showed action against *P. larvae* and the antimicrobial activity was verified by the ability to cause rupture of the bacterial plasma membrane and consequent release of cytoplasmic constituents [70]. 

The results showed that the tested products caused damage to the bacterial membrane and resulted in the inhibition of bacterial growth of both bacteria, which allows us to consider them as potential natural alternatives for the control of pathogens such as those responsible for diseases in *A. mellifera* larvae or even in other bee species.

## Conclusions

The EEP U+U extraction method proved to be advantageous due to the shorter EEP preparation time and the highest content of total phenolic compounds, because there were no significant differences among the extraction methods and antibacterial activities.

Melittin and nisin were efficient in vegetative and sporulated forms of *P. alvei* and *B. subtilis*, with the highest inhibitory and bactericidal activities for both bacteria. Cinnamaldehyde showed a better action on *P. alvei*. 

*B. subtilis* and *P. alvei* exposed to all the antimicrobials tested showed a gradual increase in the concentration of proteins released over time, the release of absorbable cellular constituents at 260 nm, phosphate and potassium ions, indicating damage to the bacterial cell membrane.

 All products studied showed antibacterial activities and synergism in association with tetracycline, whether bactericidal or bacteriostatic, indicating potential therapeutic alternatives against *P. alvei* and *B. subtilis* for the control of diseases in bee larvae.

### Abbreviations

AFB: American foulbrood; AMPs: antimicrobial peptides; ATCC: American Type Culture Collection; BHI: brain heart infusion; CFU: colony-forming units; DPPH: 2,2-diphenyl-1-picryl-hydrazyl; EEP: ethanol extract of propolis; EFB: European foulbrood; MIC: minimum inhibitory concentration; MBC: minimum bactericidal concentration; IU: international unit; KCl: potassium chloride; M+M: double maceration; M+U: maceration associated with ultrasonication; M_n_Cl_2_: manganese chloride; OD: optical density; PBS: phosphate-buffered saline; REMA: resazurin microtiter assay plate; U+U: double ultrasonication; UV: ultraviolet. 
